# Deciphering the Topology of Sitagliptin Using an Integrated Approach

**DOI:** 10.1021/acsomega.4c09930

**Published:** 2025-01-10

**Authors:** Renny Mathew, Brijith Thomas

**Affiliations:** Science Division, New York University Abu Dhabi, P.O. Box 129188, Abu Dhabi, United Arab Emirates

## Abstract

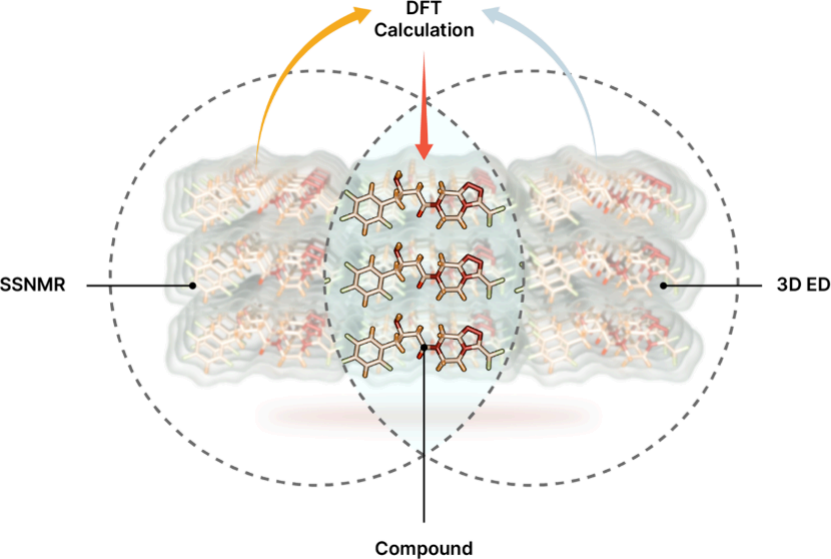

Determining the structure of sitagliptin is crucial for ensuring its effectiveness and safety as a DPP-4 inhibitor used to treat type 2 diabetes. Accurate structure determination is vital for both drug development and maintaining quality control in manufacturing. This study integrates the advanced techniques of solid-state nuclear magnetic resonance (NMR) spectroscopy, three-dimensional (3D) electron diffraction, and density functional theory (DFT) calculations to investigate the structural intricacies of sitagliptin. Solid-state NMR provides detailed information on the molecular environment, revealing insights into the atomic-level structure. The DFT calculations complement these experimental findings by offering theoretical insights into the electronic structure and helping validate the NMR data. Dynamic nuclear polarization has recently emerged as a cornerstone approach to enhance the sensitivity of solid-state NMR spectroscopy under magic angle spinning (MAS), opening unprecedented analytical opportunities. In this work, we incorporated the latest state-of-the art dynamic nuclear polarization NMR into 3D ED NMR crystallography. The findings from this study have important implications for the pharmaceutical industry, particularly in enhancing the precision of drug development and ensuring the high quality of diabetes treatments. Overall, this combined methodological approach not only advances the structural characterization of sitagliptin but also sets a precedent for analyzing other pharmaceutical compounds of similar complexity.

## Introduction

Sitagliptin, a selective DPP-4 inhibitor approved by the FDA in 2006, is a key treatment for type 2 diabetes, enhancing incretin hormones to improve insulin secretion and reduce hepatic glucose production. Sitagliptin is well tolerated, does not cause weight gain or increase the risk of hypoglycemia, and supports cardiovascular health.^1,2^ Its once-daily oral dosage promotes adherence while also potentially improving pancreatic beta-cell function. As diabetes rates rise globally, sitagliptin’s role in disease management becomes increasingly significant.^3,4^

Despite its significant therapeutic benefits and widespread use, there remains a gap in the comprehensive structural understanding of sitagliptin, particularly in its anhydrous form. Knowing the structure of the pharmaceutical molecule is essential to explain the structure–property relationship. The phosphate salt form of sitagliptin is well characterized and commonly utilized in clinical settings, but the structure of sitagliptin without phosphate remains elusive.^2^ Single-crystal XRD provides a 3D view of the molecule, offering insights into the spatial arrangement of atoms and the intricate bonding interactions that define its stability and reactivity. The difficulty in obtaining single crystals of sitagliptin without its phosphate counterpart hinders the ability to fully understand its structure using a conventional approach. Without this information, certain aspects of sitagliptin’s behavior, particularly those related to its pure, unbound form, remain challenging.

While conventional techniques such as X-ray crystallography or neutron diffraction are highly effective in determining the three-dimensional arrangement of atoms in crystals, they face difficulties when dealing with crystals that exhibit disorder. To address this gap, an interdisciplinary approach using solid-state NMR spectroscopy, 3D ED,^5−7^ and density functional theory (DFT) calculations is employed.^8−22^ These techniques can provide complementary insights into situations where conventional XRD is limited. Solid-state NMR plays a central role in capturing intricate details concerning the bonding network within a material while also providing valuable insights into the proximities and precise distances between various sites within the structure.^23^ Consequently, it stands out as an indispensable method for characterizing complex materials at the atomic level, particularly those exhibiting disorder and heterogeneity. High-field dynamic nuclear polarization (DNP)^24−26^ combined with low-temperature magic angle spinning (MAS) has significantly enhanced the sensitivity of solid-state NMR experiments, achieving improvements by several orders of magnitude. This technique has been recently employed to characterize materials and biological systems, and it has been successfully integrated into the NMR crystallography approach, now referred to as DNP NMR crystallography.^18,19,27−29^ The uncertainties arising from the assignment of carbon, nitrogen, and oxygen atoms, as well as the absence or ambiguity of hydrogen atoms in 3D ED measurements, could be addressed by utilizing solid-state NMR in combination with quantum mechanical calculation based on NMR crystallography.^30^ Recently, we successfully demonstrated the above-mentioned approach to determine the structure of a less ordered molecular system as illustrated in the case of disordered NO_2_–PDI and DHICA molecules.^20,21^ In short, the 3D ED NMR crystallography integrates NMR spectroscopy with 3D ED and other complementary techniques along with DFT calculations to unravel the atomic-level structure of less ordered materials.^14,31−40^

Sitagliptin has revolutionized type 2 diabetes management through its unique mechanism and safety profile. However, the complete structural characterization of its anhydrous form remains challenging. Advanced solid-state NMR, 3D ED, and DFT calculations are crucial in overcoming this hurdle, enhancing our understanding of sitagliptin structure, and advancing pharmaceutical sciences for improved diabetes treatments.

## Results and Discussion

**Figure 1:**
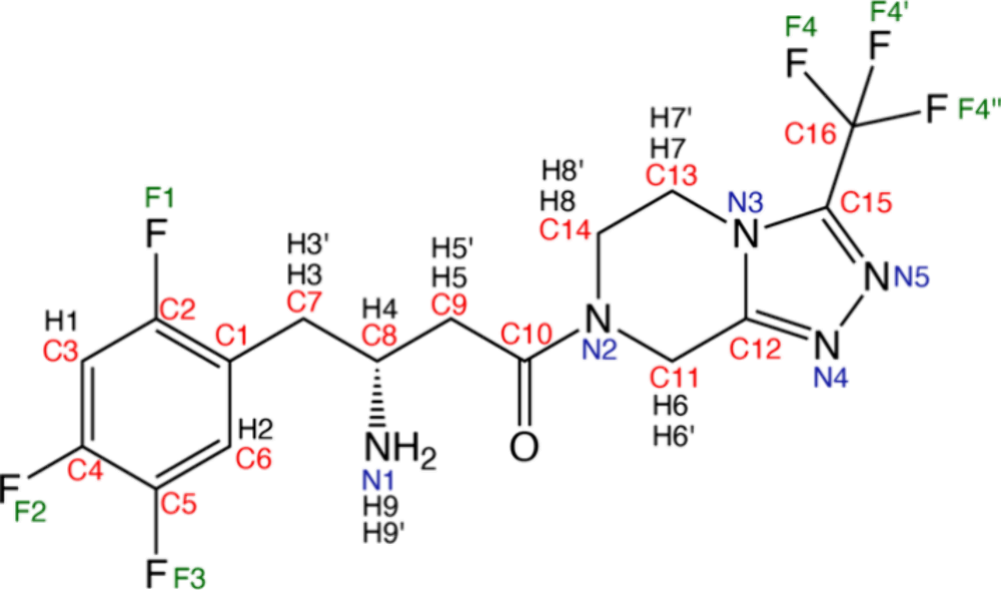
Structure of sitaglipin with the numbering scheme used and it consists of dipeptidyl peptidase-4, triazolopyrazine ring, trifluoromethyl, and trifluorophenyl groups.

The core structure of sitagliptin is based on a triazolopyrazine ring, which is critical for its biological activity (Figure 1). This heterocyclic component is essential for binding to the DPP-4 enzyme. At one end, sitagliptin features a trifluorophenyl group, where the fluorine atoms enhance the drug’s metabolic stability and increase its binding affinity for the DPP-4 enzyme. The molecule also contains an amine group, crucial for its pharmacological activity, which interacts with key residues within the enzyme’s active site. The amine group, attached to the triazolopyrazine ring, increases the molecule’s lipophilicity, influencing its distribution and duration of action in the body. Additionally, the butan-2-amine side chain plays a significant role in ensuring the proper orientation of the molecule during its binding to the DPP-4 enzyme.

### 3D ED Structure

**Figure 2:**
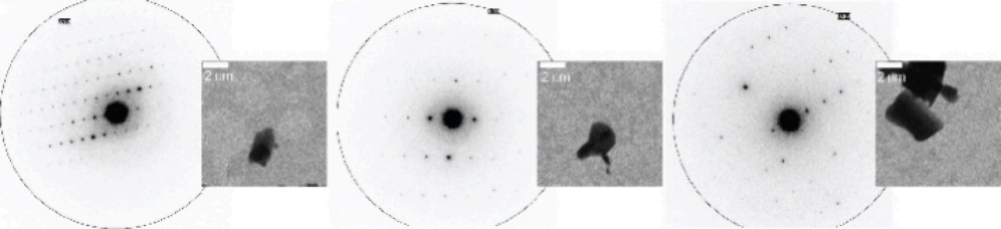
Selected TEM images and electron diffraction patterns obtained for sitagliptin at different orientations.

The microcrystalline powder was suitable for 3D ED (Figure 2 and Figure S1 and Table S2). Data were recorded on an ELDICO ED-1 electron diffractometer and analyzed, as further detailed in the Supporting Information. Sitagliptin crystallizes in the *P*_21_ space group *a* = 11.97 Å, *b* = 5.99 Å, *c* = 12.23 Å, α = 90.00°, β = 100.95°, and γ = 90.00° with two molecules in the asymmetric unit (Table 1). A reasonable *R* factor below 20 was achieved.

**Table 1:** Crystallographic Details of 3D ED Kinematic Refinement against Combined Data

**parameters**	**sitagliptin**
*a*, *b*, *c* (Å)	11.97(13), 5.99(7), 12.23(14)
α, β, γ (°)	90, 100.95(4), 90
space group	*P*_21_
formula	C_16_H_15_F_6_N_5_O
independent reflections	2123
parameters	297
restraints (non-H)	570
resolution (Å)	0.90
completeness (%)	87.6
*R*_int_ (%)	15.13
*R*_1_ [*l* > 2σ(*l*)] (%)	14.55
*wR*_2_ [all data] (%)	40.33
goodness of fit	1.07

The sample exhibits sensitivity to the electron beam primarily due to the presence of the aliphatic chain in the system. The stacking is stabilized by intermolecular hydrogen bonding between the NH_2_ and carbonyl groups on adjacent molecules. Two types of disorder are observed in the electron diffraction data, which are as follows: (i) the mobility of the CF_3_ group (ii) and the different orientations of the NH_2_ group. To capture the anomalies in 3D ED measurements, a library of structures was constructed followed by solid-state NMR combined with DFT calculations.

A suite of multinuclear solid-state NMR experiments was performed on ^1^H, ^13^C, ^15^N, and ^19^F nuclei.^2^ The DNP enhanced solid-state NMR spectra were collected to enhance the sensitivity for the low-abundance nuclei ^13^C (na 1.07%) and ^15^N (na 0.364%).

### ^1^H NMR Measurement

**Figure 3:**
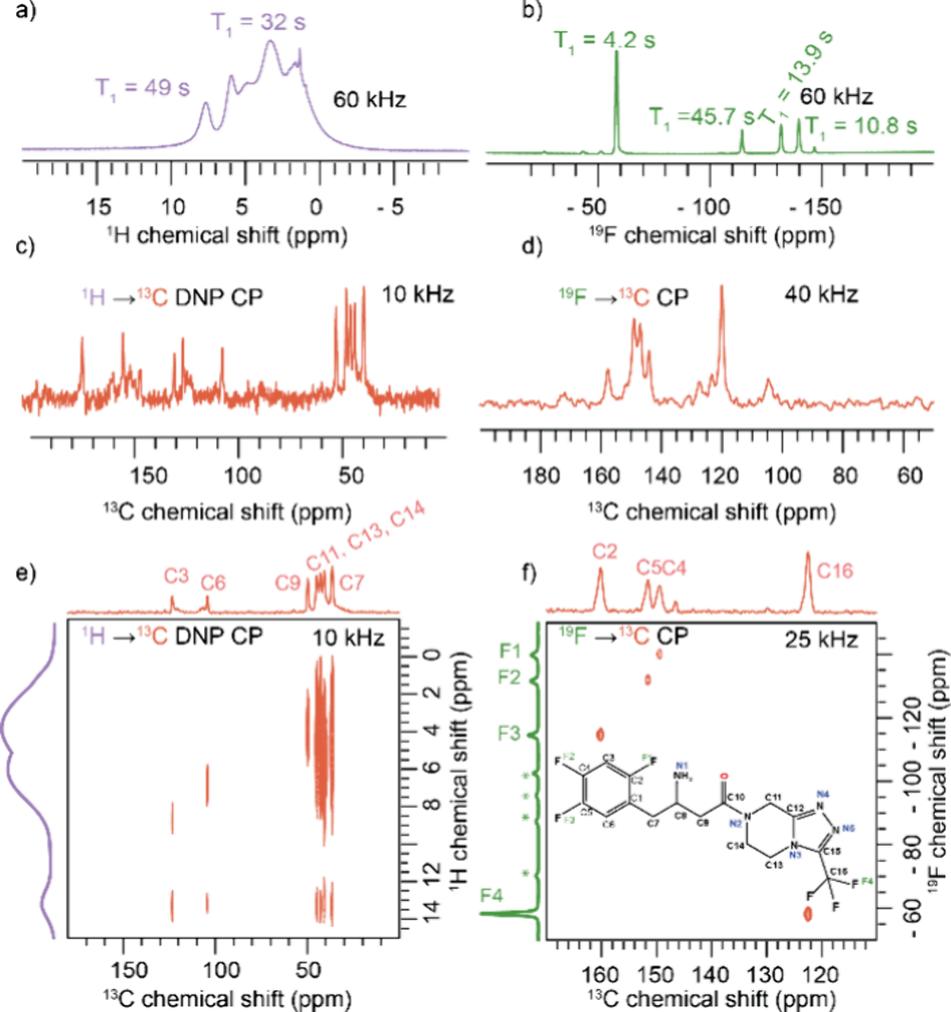
(a) The ^1^H NMR spectra (b) and the ^19^F spectra were collected at a spinning speed of 60 kHz at room temperature. In the ^19^F spectra, four sets of peaks are distinctly observed, corresponding to the CF_3_ and aromatic CF groups. The spinning side bands are indicated by asterisks. (c) The one-dimensional ^1^H → ^13^C CP spectra with MW ON are shown. (d) The ^19^F–^13^C CP spectra were collected at a spinning speed of 25 kHz and a temperature of −20 °C. (e) The ^1^H → ^13^C HETCOR spectra with MW ON of the sample at short contact times provide information about the CH species. Two sets of aromatic carbon peaks are observed in the ^1^H → ^13^C HETCOR short contact time. (f) ^19^F → ^13^C HETCOR spectra collected at 25 kHz spinning speed. All the spectra were collected at a magnetic field strength of 14.1 T.

Proton spectra collected at 60 kHz for sitagliptin reveal the three main sets of protons: aromatic, aliphatic (including alkyl and cycloalkyl), and NH_2_ protons. The protons on the amine group show a peak around 9.0 ppm (Figure 3 and Figures S2 and S3).

### ^1^H–^13^C HETCOR

The ^1^H → ^13^C HETCOR spectrum provides correlation data between carbon and hydrogen atoms, specifically identifying carbons directly bonded to protons by utilizing a short contact time. Here, we utilized dynamic nuclear polarization (DNP) solid-state NMR using the AsymPolPOK radical to get the heteronuclear correlation spectra (Figure 3 and Figures S4 and S5). An enhancement of 8 is observed in the ^13^C CP spectra with the MW ON condition (Figure S5). The ^1^H–^13^C HETCOR spectrum reveals peaks corresponding to aliphatic, aromatic, and cycloalkyl ring structures. The peaks observed at 103.8 and 122.6 ppm correspond to C3 and C6, respectively. The aliphatic peaks of 36.7, 49.7, and 45.1 ppm correspond to C7, C8, and C9 carbons, respectively. The C13, C11, and C14 carbons on the cycloalkyl groups were assigned to 42.1, 43.2, and 37.5 ppm, respectively, based on the DFT calculation. Additionally, correlations between −NH and various aliphatic and aromatic carbons C3 (103.8 ppm), C7 (36.7 ppm), and C9 (45.1 ppm) were observed in the DNP HETCOR spectra at a low temperature of 100 K.

### ^19^F NMR Measurement

Fluorine-19 (^19^F) is a spin −1/2 nucleus with a natural abundance of 100% and the second most sensitive stable NMR-active nucleus with a signal sensitivity of 83.4% relative to ^1^H. Unlike hydrogen, which is typically surrounded by a single electron, the fluorine nucleus is generally surrounded by nine electrons. This results in a significantly higher range and sensitivity of chemical shifts to the local environment for fluorine compared to hydrogen. Consequently, ^19^F NMR has unique applications, especially in NMR crystallography.^41^ In our ^19^F NMR study of sitagliptin, we identified four distinct fluorine peaks arising from aliphatic and aromatic fluorine atoms (Figure 3). The fluorine on the trifluoro methyl group (F4) appears at −57.9 ppm. Meanwhile, the fluorine attached to aromatic carbon appears at (F3) −139.9 ppm, (F2) −131.9 ppm, and (F1) −114.6 ppm. The longitudinal relaxation time (T_1_) of the aliphatic fluorine was found to be short, around 4.2 s, a result attributed to the mobility of the CF_3_ group.^29,30^ F3 has the longest spin–lattice relaxation around 45.7 s, which might be due to the stacking of the aromatic moieties leading to the rigidity.

### ^19^F → ^13^C HETCOR

The initial ^13^C and ^19^F chemical shift assignments done with the aid of 1D experiments were further validated and refined using 2D ^13^C–^1^H and ^13^C–^19^F HETCOR experiments (Figure 3). The ^19^F–^13^C HETCOR spectra at short contact time provide information about carbons directly bonded to fluorine, as evidenced by the observation of four distinct correlation peak sets corresponding to these carbons. A correlation is observed between (C16) 122.6 ppm and (F4) −57.9 ppm, which corresponds to the trifluoromethyl group. A correlation is observed between (C5) 149.9 ppm and (F3) −139.9 ppm, which corresponds to the −CF group. Similarly, a correlation between (C4) 151.7 ppm and (F2) −131.9 ppm is observed corresponding to fluorine attached to aromatic carbon. In addition, a correlation between (C2) 160.2 ppm and (F1) −114.6 ppm is observed.

### HETCOR Long Contact Time

**Figure 4:**
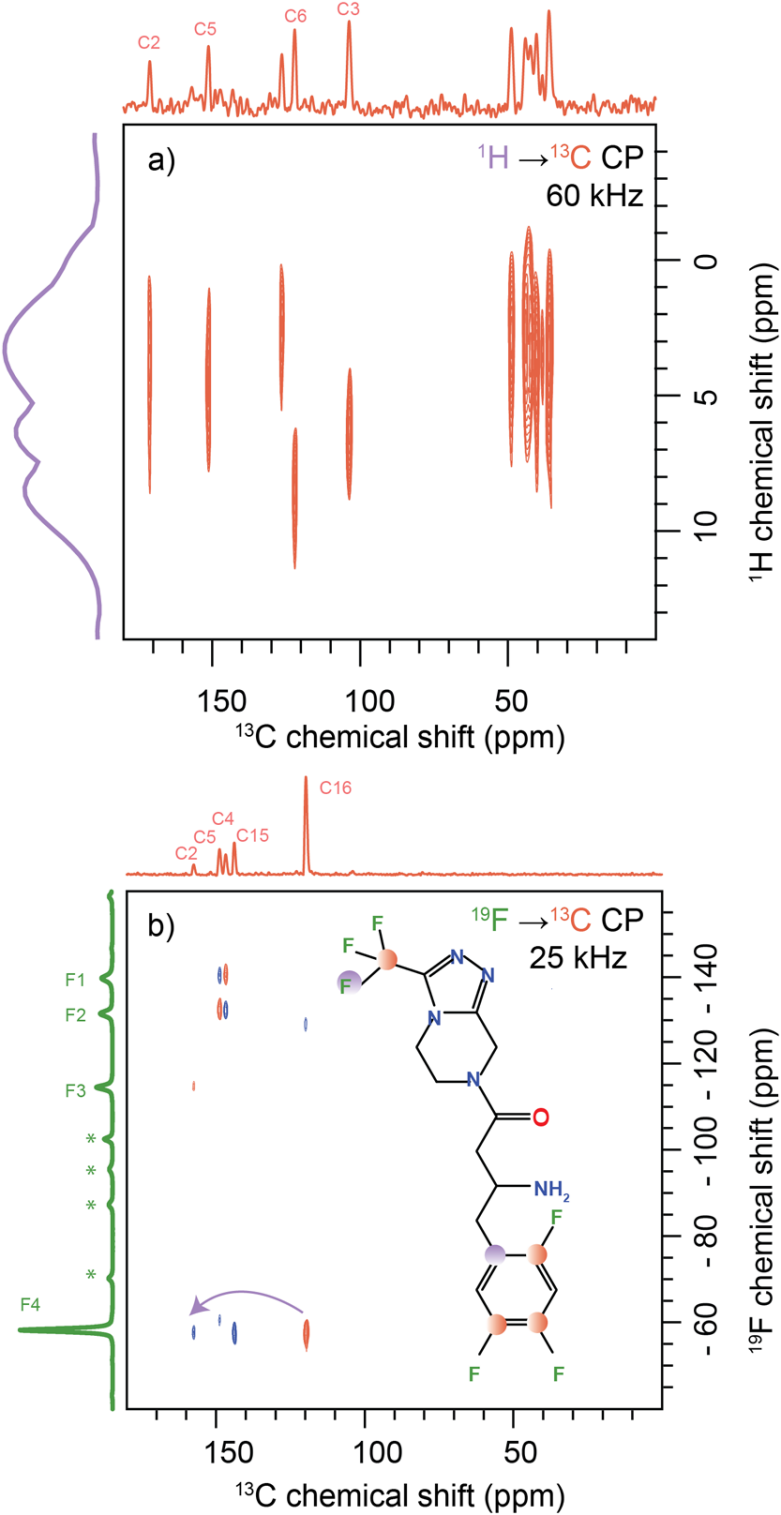
(a) ^1^H → ^13^C and (b) ^19^F → ^13^C HETCOR spectra of sitagliptin collected at long contact time, revealing both intermolecular and intramolecular correlations. In the ^1^H → ^13^C HETCOR spectrum, correlations between the CH_2_ group and aromatic carbons are clearly observed at long contact times.

Solid-state NMR experiments, particularly heteronuclear correlations with long contact times, are used to discern intermolecular correlations that are important for understanding molecular packing. The ^19^F → ^13^C HETCOR spectra, acquired with a long contact time of 7 ms, provide insights into intermolecular correlations (Figure 4).

An intramolecular correlation between F4 and C16 is observed. On a similar line, correlation between F4 and quaternary carbon C15 is observed, which is also intramolecular. A correlation between F4 and C2, C4, and C5 is observed, which should be intermolecular. The above-mentioned intermolecular interaction points toward the head-to-tail arrangement of molecules in the packing. A new correlation peak is observed between F3 and C2, which is within the aromatic ring and primarily intramolecular. A correlation from F1 and F2 with C4 and C5 is also observed, which are intramolecular. In ^1^H → ^13^C HETCOR spectra with long contact times, correlation of the aliphatic protons with C1, C2, and C5 is observed, which could be inter- or intramolecular.

### ^15^N Measurements

**Figure 5:**
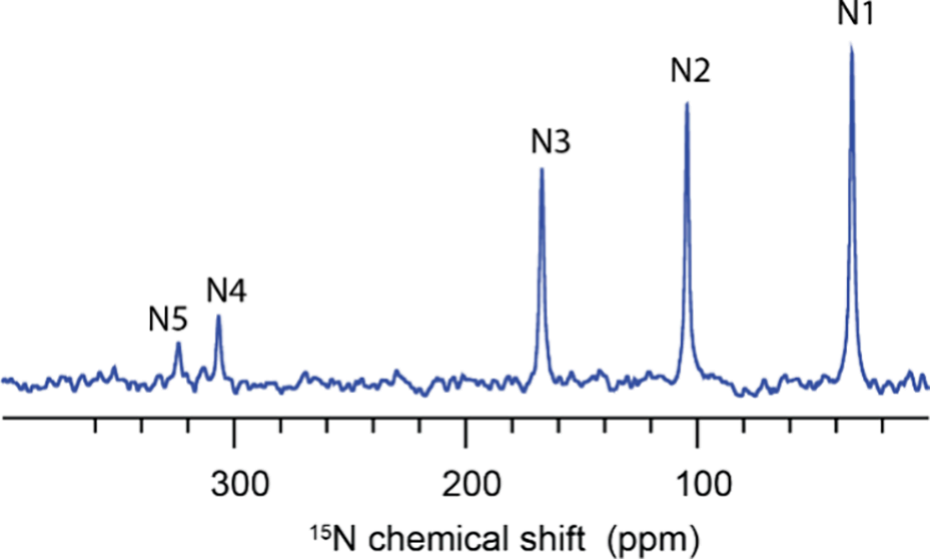
DNP enhanced ^15^N CP spectra of sitagliptin B obtained with the AsymPolPOK radical at 100 K.

The five sets of nitrogen peaks were observed in the CP experiment with chemical shifts of 33.1, 104.2, 167.0, 306.5, and 324.0 ppm corresponding to N1, N2, N3, N4, and N5, respectively (Figure 5). The chemical shifts are in line with the previously reported values of the sitagliptin phosphate molecule.

### DFT Calculations

Based on 3D ED data, four different models were constructed to address the challenge with understanding about the orientation of the −NH_2_ and −CF_3_ groups. Cases A and B are based on the orientation of the −CF_3_ group (Figures S6 and S7). Cases C and D are based on the orientation of the NH_2_ group. The four different models were analyzed using the correlation information obtained in the heteronuclear correlation experiment at long contact time, the simulated chemical shift data from the DFT calculation, and the calculated energy. Cases C and D are less stable energetically due to the lack of intermolecular hydrogen bonding. Chemical shift information was used to evaluate and compare four different models constructed from the 3D ED data. RMSDs of 3.5 ppm in the case of ^13^C and 3.0 ppm in the case of ^19^F are obtained for case A, as shown in Table 1. Case A, which gives the lowest RMSD, is the most stable structure and is mainly stabilized by intermolecular hydrogen bonding (Table S5). For the ^13^C chemical shift, RMSDs of 3.5 ppm in cases A and B and 3.6 ppm for cases C and D are obtained (Table 2). In the case of the ^19^F chemical shift, calculated RMSDs between the experiment and simulation are as follows: 3.0 ppm in the case of A, 2.9 ppm in the case of B, 3.3 ppm in the case of C, and 3.2 ppm in the case of D.

**Table 2:** Comparison of the Relative Energies and ^13^C RMSD Values Derived from Four Different Possible Structures Obtained through 3D ED

**label**	**relative energy** (kcal/mol)	**^**13**^**C RMSD** (ppm)**	**^**19**^**F RMSD** (ppm)**
case A	0.00	3.6	3.0
case B	–0.02	3.6	2.9
case C	0.058	4.0	3.3
case D	0.57	4.0	3.2

Additionally, the powder X-ray diffraction (PXRD) pattern of sitagliptin was collected and compared with the simulated PXRD pattern (Figure S8), demonstrating a close match between the experimental and simulated patterns.

### Crystal Architecture

**Figure 6:**
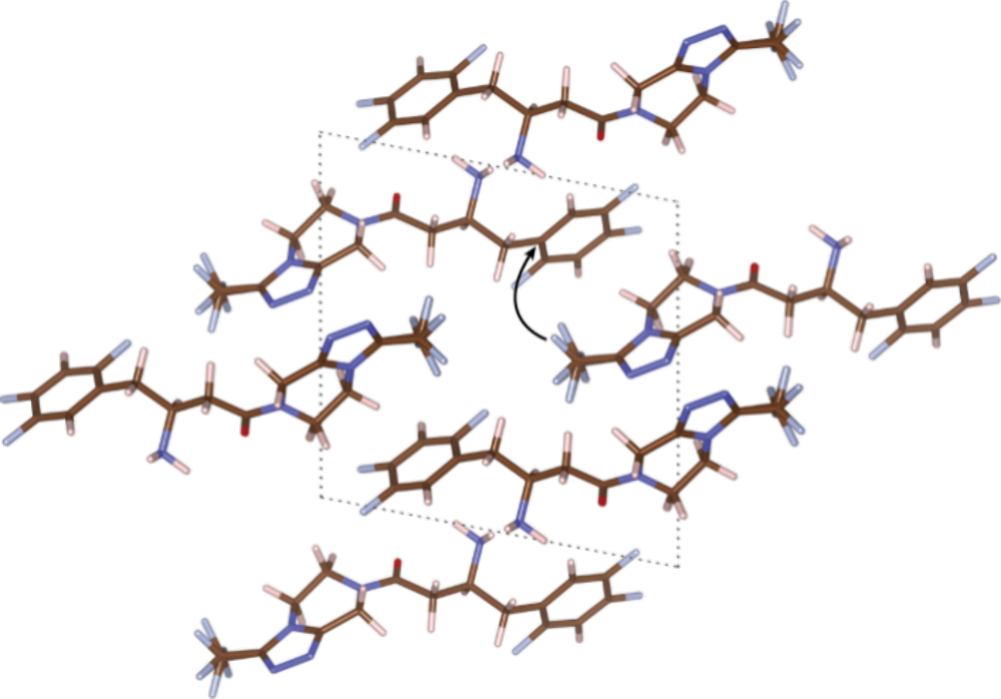
Proposed crystal structure of the sitagliptin molecule is characterized by unit cell parameters: *a* = 11.97 Å, *b* = 5.99 Å, and *c* = 12.23 Å, with angles α = 90.00°, β = 100.95°, and γ = 90.00°.

The sitagliptin stacks in the *P*_21_ space group with two molecules in the unit cell. The monomer unit is arranged in such a way that the imidazole ring is perpendicular to the aromatic part, a configuration resulting from the twist caused by the aliphatic bridge (Figure 6 and Figures S9–S11). The molecule stacks along the *c* axis. The stabilization of the packing is attributed to hydrogen bonding interactions, and it is clearly evident from the energies of cases A and B. Hydrogen bonds form between protons on the NH_2_ group and oxygen atoms on adjacent molecules. The orientation of the NH_2_ group is influenced by both intramolecular hydrogen bonding with a nearby oxygen atom and intermolecular hydrogen bonding with the NH_2_ group of a neighboring molecule. Two types of hydrogen bonding are present in the crystal packing: (i) intramolecular hydrogen bonding between protons on NH_2_ (H9 and H9′) and oxygen corresponds to a 2.425 Å distance; (ii) intermolecular hydrogen bonding between protons on the NH (H9 and H9′) with the adjacent nitrogen group (N1) on the nearby molecule and the distance corresponds to 2.32 Å. The head-to-tail arrangement of sitagliptin molecules is primarily driven by these hydrogen bonding interactions. Each independent molecule is interconnected through extensive hydrogen bonding, forming chains. Cases A and B, which have the lowest energies, exhibit hydrogen bonding. Among the four different cases, case D, where the orientation of the NH bond is rotated outward, exhibits the highest energy. This is due to the absence of intermolecular hydrogen bonding (Figure S10).

The disorder in the CF_3_ group arises from mobility, and it is evident from the T_1_ measurements also. The T_1_ measurements of ^19^F and ^1^H provide valuable insights into the dynamics of aliphatic and aromatic moieties. The shorter T_1_ relaxation times around 4.2 s, times indicating faster relaxation, attributed to the dynamic nature of the CF_3_ groups, leading to the averaging of ^19^F chemical shifts. The rapid motion of the fluorine atoms in the CF_3_ groups produces a single sharp peak in the NMR spectrum due to this averaging effect.

## Conclusions

In conclusion, our study offers a significant understanding of the structure of sitagliptin by employing an integrated approach that includes solid-state NMR spectroscopy, density functional theory calculations, dynamic nuclear polarization solid-state NMR, and 3D electron diffraction. A library of structures was generated from 3D electron diffraction (ED) studies, and the results showed favorable agreement between the simulated and observed NMR parameters. This interdisciplinary methodology has allowed us to unravel the complex hydrogen bonding network and molecular interactions that stabilize sitagliptin’s structure, particularly in its anhydrous form, providing insights that were previously unattainable with conventional techniques. The methodologies developed in this study have the potential to advance the characterization of other complex pharmaceutical compounds, ultimately contributing to the precision and efficacy of drug development.

## Methods

### Materials and Sample Preparation

Samples were obtained from Sigma-Aldrich and used as received.

### Solid-State NMR

Solid-state NMR spectra were recorded by using a Bruker Avance HD 600 WB spectrometer. For DNP experiments, measurements were conducted on a 14.1 T Bruker AVANCE III system with a 395 GHz gyrotron and a 3.2 mm MAS probe. The spinning speed was 10 kHz, and the sample temperature was ∼100 K. Experiments included cross-polarization steps for ^13^C and ^15^N measurements. Higher-speed MAS experiments at 25 and 60 kHz were performed with detailed parameter optimization, including ramped CP, SPINAL64 decoupling, and DUMBO schemes. Calibration used alanine and adamantane standards (see the Supporting Information).

### DNP Solid-State NMR

Sitagliptin samples for DNP NMR experiments were impregnated with a 10 mM solution of the AsymPolPOK biradical in D_2_O (∼40 mg ground with 60 μL of solution) and packed into a 3.2 mm sapphire rotor for analysis (see the Supporting Information).

### Solution-State NMR

^1^H and ^13^C NMR spectra in solution were measured on a 500 MHz Bruker Advance DPX spectrometer with TMS as an internal standard.

### DFT Calculations

Structural optimizations were performed using the FORCITE module in Materials Studio with the Universal force field followed by plane-wave DFT calculations using CASTEP. The PBESOL functional and dispersion corrections (Tkatchenko–Scheffler method) were employed. An energy cutoff of 630 eV and a Monkhorst–Pack grid with 0.07 Å^–1^ spacing ensured calculation efficiency (see the Supporting Information).

### PXRD and 3D Electron Diffraction

Powder X-ray diffraction (PXRD) was conducted with a Rigaku Smartlab diffractometer (Cu Kα radiation). For 3D electron diffraction, samples were analyzed using an ELDICO ED-1 diffractometer with a LaB_6_ electron source (160 kV). Data processing utilized APEX4, SAINT, and SADABS software for structural refinement, and crystallographic data were validated using ShelXT and ShelXL. Supplementary data are deposited under deposition number 2389054 (www.ccdc.cam.ac.uk/structures) (see the Supporting Information).

## Supporting Information Available

Additional solid-state NMR spectra, comparison of experimental and plane-wave DFT-calculated ^13^C and ^19^F NMR parameters, TEM images, PXRD pattern, and images of structural models (PDF)
CIF file of the data used in this work (CIF)

## Supplementary Material

ao4c09930_si_001.pdf

ao4c09930_si_002.cif
