# Supplementary material for: Deciphering the Topology of Sitagliptin Using an Integrated Approach
Source: ACS Omega. 2025 Jan 10;10(2):2289–95. doi: 10.1021/acsomega.4c09930 (PMC11755177; doi:10.1021/acsomega.4c09930)
Supplement: Supplementary file 1 — ao4c09930_si_001.pdf [file ao4c09930_si_001.pdf]

Deciphering the Topology of Sitagliptin Using an Integrated Approach

Renny Mathew[a], Brijith Thomas[a]*

[a]Science Division, New York University Abu Dhabi, P.O. Box 129188, Abu Dhabi, United Arab Emirates; E-mail: brijiththomas@nyu.edu

Table of Contents

Section 1. Materials and Methods 2

Section 2: Tables 4

Table S1: Comparison of the state of the art in the combination of 3D ED and solid-state NMR approach. 4
Table S2: 3D ED data collection details for the Sitagliptin crystals....................4
Table S3: Comparison of energy for cases A, B, C and D 4
Table S4: The predicted ^{13}C NMR chemical shift and root mean square deviation for case A 5
Table S5: The predicted ^{13}C NMR chemical shift and root mean square deviation for case B....................5
Table S6: The predicted ^{13}C NMR chemical shift and root mean square deviation for case C....................6
Table S7: The predicted ^{13}C NMR chemical shift and root mean square deviation for case D 6
Table S8: The predicted ^{19}F NMR chemical shift and root mean square deviation for case A....................7
Table S9: The predicted ^{19}F NMR chemical shift and root mean square deviation for case B 7
Table S10: The predicted ^{19}F NMR chemical shift and root mean square deviation for case C 7
Table S11: The predicted ^{19}F NMR chemical shift and root mean square deviation for case D....................8

Section 3: Figures....................8

Figure S1: The TEM image of the sitagliptin molecule… 8
Figure S2: The solution state ^{1}H NMR spectra of the sitagliptin molecule....................10
Figure S3: The ^{1}H-^{1}H BABA spectra of the sitagliptin molecule 10
Figure S4. The solution state ^{13}C NMR spectra of the sitagliptin molecule....................11
Figure S5: The DNP MW ON and OFF spectra....................11
Figure S6: The four different cases of sitagliptin molecule....................12
Figure S7: The orientation of CF_3 group 12
Figure S8: The comparison of PXRD pattern....................13
Figure S9: The structure showing inter and intra molecular packing in sitagliptin molecule 13
Figure S10: The structure showing inter and intra molecular packing in sitagliptin molecule 14
Figure S11: The Schematic diagram....................14
Figure S12: The ^{1}H magnetization build up curves obtained using saturation recovery experiment at 60 kHz spinning speed 14
Figure S13: The ^{19}F magnetization build up curves obtained using saturation recovery experiment at 60 kHz spinning speed 15

Section 1: Materials and Methods

Material: The samples were obtained from the Sigma Aldrich and used as obtained.

Solid-State NMR: All solid-state NMR spectra were measured with a Bruker Avance HD 600 WB NMR spectrometer ($\nu_0(^1H)$ = 600.5 MHz).

For DNP NMR experiments, sitagliptin samples were impregnated with a solution of 10 mM solution of AsymPol-POK [1] biradical in D_2O (~40mg ground w/ 60uL of 10mM cAsymPol-POK in D_2O). The sample has a pasty consistency and packed into a 3.2 mm sapphire rotor. All DNP experiments were performed on 14.1 T Bruker AVANCE III wide-bore NMR system equipped with a 395 GHz gyrotron, a transmission line, and a low temperature (~100 K) triple resonance 3.2 mm MAS probe head. The DNP enhanced ^1H signal is transferred through a cross-polarization step before any ^{13}C and ^{15}N experiments. All the solid-state NMR spectra were recorded at a spinning frequency of 10 kHz with a recycle delay of 5s, and the sample temperature was regulated at ~100 K.

Experiments at 24 kHz: The fine powder of the samples was packed in a 3.2 mm outer diameter (o. d.) ZrO_2 rotor. For all experiments, the MAS speed was 24 kHz. The ^1H-^{13}C ramp-cross-polarization (rampCP)[2] experiments were performed with a ^{13}C nutation frequency of 60 kHz, and the ^1H nutation frequency optimized at the +1 Hartman-Hahn condition[3] ~84 kHz. The repetition delay was set to 7 s. $^1H\{^{13}C\}$ CP-HETCOR experiments were performed with a contact time of 50 μs and 1.0 ms, and 32-128 t_1 increments consisting of 256-512 scans were collected in each experiment. SPINAL64 decoupling[4] (^1H B_1 field of ~83 kHz) was applied during acquisition. DUMBO[5] homonuclear decoupling schemes were employed during the t_1 evolution at ~100 kHz. The ^1H scaling factor for DUMBO HETCOR was corrected using alanine as the standard, and ^{13}C shift scale were calibrated with adamantane as an external standard.

Experiments at 60 kHz: Single-pulse ^1H spectra were recorded using 90° rf pulses operating at the ^1H nutation frequency $\nu_H \approx 185$ kHz, 16 accumulated NMR-signal transients, and relaxation delays (τ_{relax}) of 2.0 s. The ^{13}C spectra were recorded by the $^1H \rightarrow {}^{13}C$ CP at the double quantum Hartmann−Hahn condition,[3] $\nu_H + \nu_C = \nu_r$, which involved ramped CP of ν_H = 20 ± 5 kHz for ^1H (ν_C = 40 kHz), a 1.5 μs 90° ^1H pulse, and spinal-64 ^1H decoupling[6] at ν_H = 150 kHz. The $^{13}C\{^1H\}$ 2D HETCOR NMR spectra were recorded with τ_{CP} = 250 μs, and 1.0 ms τ_{relax} = 2.0 s, and dwell times of Δt_2 = 22.0 μs and $\Delta t_1 = 6\tau r$ = 100 us, where $\tau_r = \nu r^{-1}$ is the rotor period. $32(t_1) \times 2048(t_2)$ time points were collected with 2048 (τ_{CP} =1.0 ms) and 8192 (τ_{CP} = 100 μs) accumulated transients per t_1 value. The 2D NMR data sets were zero-filled to 64 t_1 points, along with 4096 (t_2), and were apodized by a $\cos^2$ and an exponential function along the indirect and direct dimensions, respectively, with the latter giving a 50 Hz full width at half-maximum (fwhm) Lorentzian broadening.

The DQ−SQ ^1H NMR correlation spectra were recorded with the 2D NMR protocol shown in Figure 1a of ref [7]. The 2Q coherence (2QC) excitation/reconversion was accomplished by the BaBa dipolar recoupling scheme that extends over one sole rotor period,[7] thereby giving the shortest 2Q excitation (τ_{exc}) and reconversion (τ_{rec}) intervals of $\tau_{exc} = \tau_{rec} = \tau_r$ = 16.66 μs. The ^1H nutation frequency was $\nu_H \approx 185$ kHz for the 90° dipolar recoupling pulses of a duration of 1.35 μs. The 2D NMR acquisitions employed τ_{relax} = 2.0 s, $64(t_1) \times 512(t_2)$ time points were acquired with dwell times of $\{\Delta t_1 = 3\tau_r; \Delta t_2 = 2.0$ μs$\}$ and 64 accumulated transients/t_1-value. The 2D data sets were zero-filled to 256 × 2048 points and apodized by an exponential 50 Hz fwhm Lorentzian broadening was applied both dimensions.

Solution state NMR

^{1}H and ^{13}C NMR in solution were measured on a 500 MHz Bruker Advance DPX spectrometer using 1,1,1,1-tetramethyl silane (TMS) as the internal standard.

DFT calculations for structure prediction: The FORCITE module in the Materials studio was used for the initial relaxation of the structures with the Universal force field. Geometry optimizations were performed using the Smart algorithm with a convergence tolerance energy of 0.0001 kcal mol^{-1} and force of 0.005 kcal Å^{-1} with a maximum number of iterations of 500. Plane-wave DFT calculations were done with the gauge-including projected augmented wave (GIPAW)[8,9] approach as implemented in the CASTEP[10] version 2017. Geometry optimization and NMR properties were calculated using the generalized gradient approximation (GGA) with the exchange-correlation PBESOL functional,[11] with On-the-Fly ultra-Pseudopotential.[12] And the Tkatchenko and Scheffler method was employed for dispersion corrections.[13] An energy cutoff of 630 eV with a Monkhorst−Pack grid[14] with a k-point spacing of 0.07 Å^{-1} was chosen to maximize the calculation efficiency.

PXRD: Powder X-ray diffraction pattern was collected using Rigaku Smartlab X-ray diffractometer with Cu Kα radiation.

3D electron diffraction: Samples were deposited on standard TEM grids (amorphous carbon on Cu) as dry powder without grinding and measured on an ELDICO *ED-1* electron diffractometer at room temperature using the software ELDIX.[15] The device is equipped with a LaB_6 electron source operating at an acceleration voltage of 160 kV (λ = 0.02851 Å) and a hybrid-pixel detector (Dectris QUADRO). The grid was screened for suitable crystals in STEM (scanning transmission electron microscopy) mode and diffraction data were recorded in continuous rotation mode with a pseudo-parallel beam of ca. 750 nm diameter. Parts of measurements affected by beam damage or shadowing by the grid were omitted. Measurement details for all datasets used are given in Table S2.

Data were processed using the APEX4 software package.[16] Frames were integrated separately for each crystal, then merged, scaled, and corrected for Lorentz effects, scan speed, background, and absorption using SAINT and SADABS.[17,18] Space group assignment was based on systematic absences, E statistics, and successful refinement of the structure. The structure was solved using ShelXT and refined with ShelXL in conjunction with ShelXle.[5,6,7] Least squares refinements were carried out within the kinematic approximation by minimizing $\Sigma w(F_{obs}{}^2\text{-}F_{calc}{}^2)^2$ with the ShelXL weighting scheme and using neutral electron scattering factors.[6,8] Non-H atoms were refined with anisotropic displacement parameters. H atoms were placed in calculated positions based on typical distances for neutron diffraction and refined as a rigid rotating group with $U_{iso}(H) = 1.5\cdot U_{eq}(N)$ for the amino group and with a standard riding model and $U_{iso}(H) = 1.2\cdot U_{eq}(C)$ for other groups. A split layer refinement was used for the disordered trifluoromethyl group and similarity restraints were used to stabilize the refinement of the layers. Deposition Number 2389054 contains the supplementary crystallographic data for this paper. These data are provided free of charge by the joint Cambridge Crystallographic Data Centre and Fachinformationszentrum Karlsruhe Access Structures service and can be accessed at www.ccdc.cam.ac.uk/structures. For comparison with NMR data, also models were created which feature only one of the two CF_3 orientations and an alternative orientation of the amino group (which gives worse refinement results) in all possible combinations.

Section 2: Tables

Table S1: Comparison of the state of the art in the combination of 3D ED and solid-state NMR approach.

Serial no	Work	Techniques used	Reference
1	PDI-NO_2	3D ED and solid-state NMR	[21]
2	Eumelanin	3D ED and solid-state NMR	[22]
3	L-Histidine	3D ED and solid-state NMR	[23]
4	L-Tyrosine	3D ED and solid-state NMR	[24]
5	PDI	3D ED and solid-state NMR	[25]
6	Sitagliptin	3D ED and solid-state NMR	This work

Table S2: 3D ED data collection details for Sitaglitpin crystals used in refinement.

Crystal no.	Approximate size [µm]	Angular range [°]	Rotation per frame [°]	Exposure time [s]	Total exposure [s]	Frames measured	Frames used
1	0.9 x 0.5 x 0.5	−60 to +60	1	1	120	120	40
2	2.0 x 1.0 x 0.8	−60 to +60	1	1	120	120	40
3	>5 x >5 x 0.9	0 to +60	1	1	60	60	40
4	2.1 x 1.1 x 0.4	−50 to +50	1	1	100	100	40
5	1.9 x 1.5 x 0.7	−40 to +40	1	1	80	80	40
6	3.1 x 2.0 x 0.8	−50 to +30	1	1	80	80	50
7	3.0 x 0.9 x 0.4	−20 to +50	1	1	70	70	40

Table S3: Comparison of energy of four different cases based on the 3D ED models.

label	Final Energy (eV)	
Case A	-17075.438	0
Case B	-17075.440	-0.002
Case C	-17075.380	0.058
Case D	-17075.381	0.57

Table S4: The predicted ^{13}C NMR chemical shift and root mean square deviation for the case A.

numbering scheme	Solution State NMR (δ ppm)	Solid state NMR (δ ppm)		CASTEP (σ ppm)	predicted chemical shift (δ ppm)
C10	170.6	171.0	-0.4	1.65	171.16
C2	156.2	160.2	-4.0	10.72	162.09
C4	150.2	149.9	-1.5	19.52	153.29

C12	143.7	154.8	-11.1	19.64	153.17
C5	149.6	151.7	-0.3	21.60	151.21
C15	149.5	146.6	2.9	28.73	144.08
C16	148.1	122.6	25.5	40.55	132.26
C1	147.5	129.5	18.0	43.43	129.38
C6	119.1	122.6	-3.5	46.95	125.86
C3	105.7	103.8	1.9	67.97	104.84
C8	45.8	49.8	-4.0	124.38	48.43
C9	40.9	45.1	-4.2	129.78	43.03
*C13	42.5	43.2	-0.7	132.94	39.87
*C11	32.1	42.1	-10.0	132.76	40.05
C7	36.2	36.7	-0.5	137.60	35.21
*C14	39.2	37.5	1.7	139.66	33.15
				RMSD	3.6

Table S5: The predicted ^{13}C NMR chemical shift and root mean square deviation for the case B.

**numbering scheme**	**Solution State NMR (δ ppm)**	**Solid state NMR (δ ppm)**		**CASTEP (σ ppm)**	**predicted chemical shift (δ ppm)**
C10	170.6	171.0	-0.4	1.71	171.10
C2	156.2	160.2	-4.0	10.64	162.17
C4	150.2	149.9	-1.5	19.51	153.30
C12	143.7	154.8	-11.1	19.60	153.21
C5	149.6	151.7	-0.3	21.60	151.21
C15	149.5	146.6	2.9	28.69	144.12
C16	148.1	122.6	25.5	40.52	132.29
C1	147.5	129.5	18.0	43.43	129.38
C6	119.1	122.6	-3.5	46.99	125.82
C3	105.7	103.8	1.9	67.98	104.83
C8	45.8	49.8	-4.0	124.40	48.41
C9	40.9	45.1	-4.2	129.76	43.05
*C13	42.5	43.2	-0.7	132.90	39.91
*C11	32.1	42.1	-10.0	132.75	40.06
C7	36.2	36.7	-0.5	137.63	35.18
*C14	39.2	37.5	1.7	139.67	33.14
				RMSD	3.6

Table S6: The predicted ^{13}C NMR chemical shift and root mean square deviation for the case C.

**numbering scheme**	**Solution State NMR (δ ppm)**	**Solid state NMR (δ ppm)**		**CASTEP (σ ppm)**	**predicted chemical shift (δ ppm)**
C10	170.6	171.0	-0.4	2.15	170.97

C2	156.2	160.2	-4.0	10.48	162.64
C4	150.2	149.9	-1.5	19.61	153.51
C12	143.7	154.8	-11.1	19.71	153.41
C5	149.6	151.7	-0.3	21.48	151.64
C15	149.5	146.6	2.9	28.59	144.53
C16	148.1	122.6	25.5	40.55	132.57
C1	147.5	129.5	18.0	43.97	129.15
C6	119.1	122.6	-3.5	47.81	125.31
C3	105.7	103.8	1.9	67.87	105.25
C8	45.8	49.8	-4.0	127.07	46.05
C9	40.9	45.1	-4.2	133.52	39.60
*C13	42.5	43.2	-0.7	132.80	40.32
*C11	32.1	42.1	-10.0	132.93	40.19
C7	36.2	36.7	-0.5	134.69	38.43
*C14	39.2	37.5	1.7	139.63	33.49
				RMSD	4.0

Table S7: The predicted ^{13}C NMR chemical shift and root mean square deviation for the case D.

**numbering scheme**	**Solution State NMR (δ ppm)**	**Solid state NMR (δ ppm)**		**CASTEP (σ ppm)**	**predicted chemical shift (δ ppm)**
C10	170.6	171.0	-0.4	2.20	170.93
C2	156.2	160.2	-4.0	10.43	162.70
C4	150.2	149.9	-1.5	19.62	153.51
C12	143.7	154.8	-11.1	19.72	153.41
C5	149.6	151.7	-0.3	21.52	151.61
C15	149.5	146.6	2.9	28.59	144.54
C16	148.1	122.6	25.5	40.56	132.57
C1	147.5	129.5	18.0	43.94	129.19
C6	119.1	122.6	-3.5	47.85	125.28
C3	105.7	103.8	1.9	67.91	105.22
C8	45.8	49.8	-4.0	127.11	46.02
C9	40.9	45.1	-4.2	133.51	39.60
*C13	42.5	43.2	-0.7	132.77	39.62
*C11	32.1	42.1	-10.0	132.92	40.36
C7	36.2	36.7	-0.5	134.69	40.21
*C14	39.2	37.5	1.7	139.62	38.44
				RMSD	4.0

Table S8: The predicted ^{19}F NMR chemical shift and root mean square deviation for the case A.

numbering scheme	Solid state NMR (δ ppm)	CASTEP (σ ppm)	predicted chemical shift (δ ppm)
F1	-114.60	255.93	-109.26
F2	-131.90	279.53	-132.86
F3	-139.90	290.43	-143.76
F4	-57.90	205.09	-58.42
		RMSD	3.0

Table S9: The predicted ^{19}F NMR chemical shift and root mean square deviation for the case B.

numbering scheme	Solid state NMR (δ ppm)	CASTEP (σ ppm)	predicted chemical shift (δ ppm)
F1	-114.60	256.08	-109.36
F2	-131.90	279.76	-133.04
F3	-139.90	290.21	-143.49
F4	-57.90	205.14	-58.42
		RMSD	2.9

Table S10: The predicted ^{19}F NMR chemical shift and root mean square deviation for the case C.

numbering scheme	Solid state NMR (δ ppm)	CASTEP (σ ppm)	predicted chemical shift (δ ppm)
F1	-114.60	254.33	-108.66
F2	-131.90	278.53	-132.86
F3	-139.90	289.63	-143.96
F4	-57.90	204.51	-58.83
		RMSD	3.3

Table S11: The predicted ^{19}F NMR chemical shift and root mean square deviation for the case D.

numbering scheme	Solid state NMR (δ ppm)	CASTEP (σ ppm)	predicted chemical shift (δ ppm)
F1	-114.60	254.40	-108.66
F2	-131.90	278.65	-132.91
F3	-139.90	289.57	-143.83
F4	-57.90	204.62	-58.89
		RMSD	3.2

Section 3: Figures

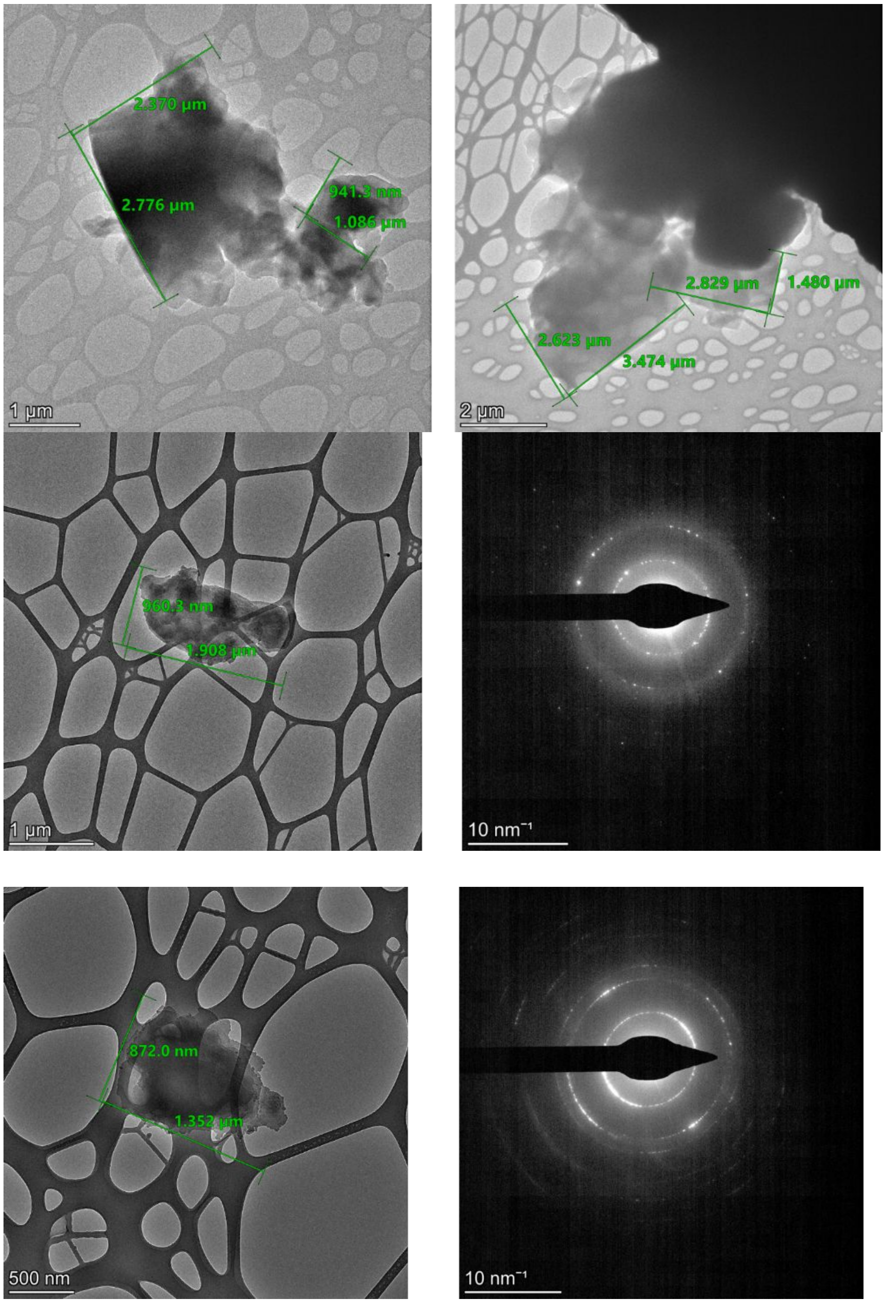

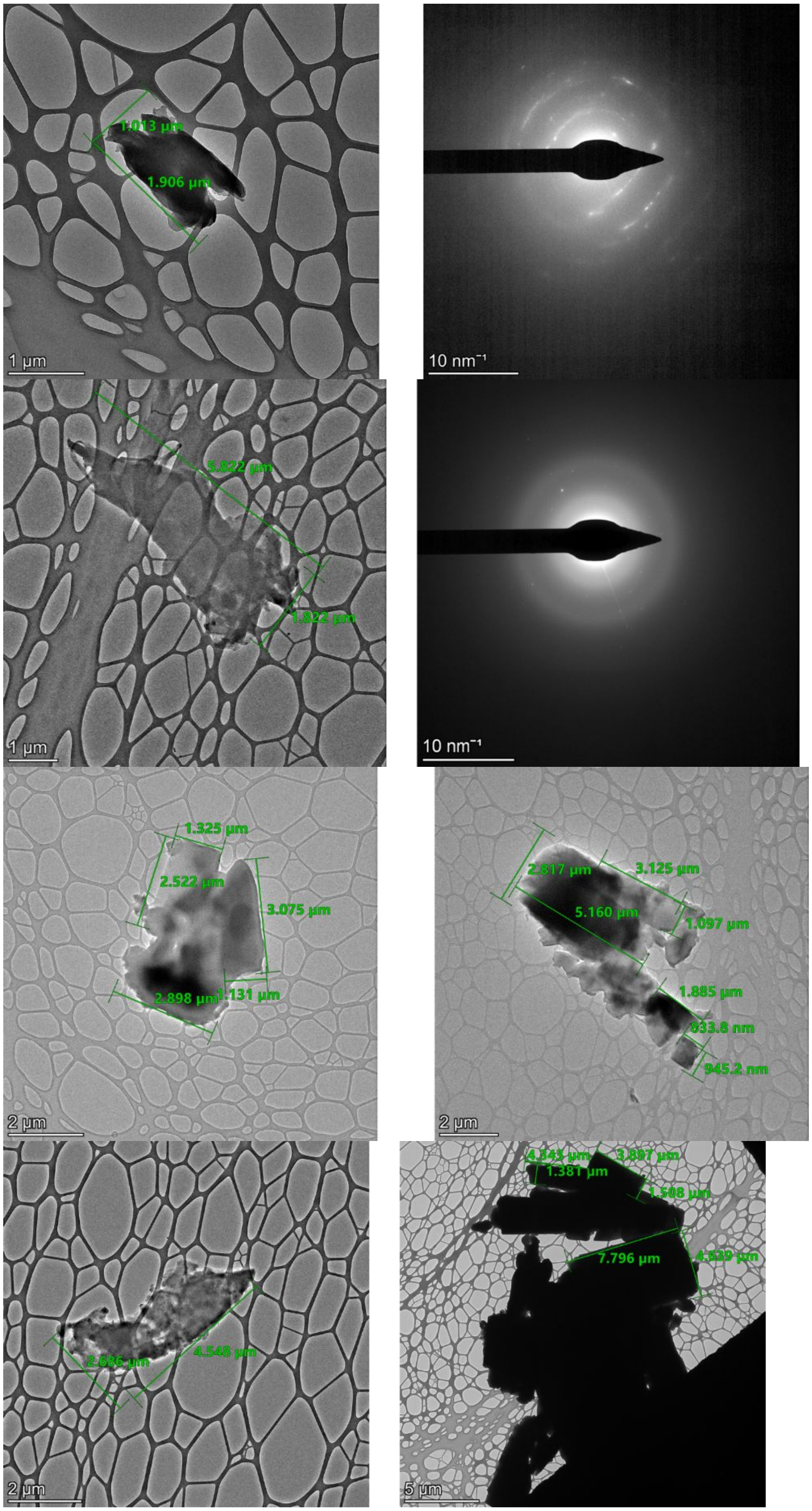

Figure S1: The TEM image of the sitagliptin molecule showing the particle size distribution collected at different orientations.

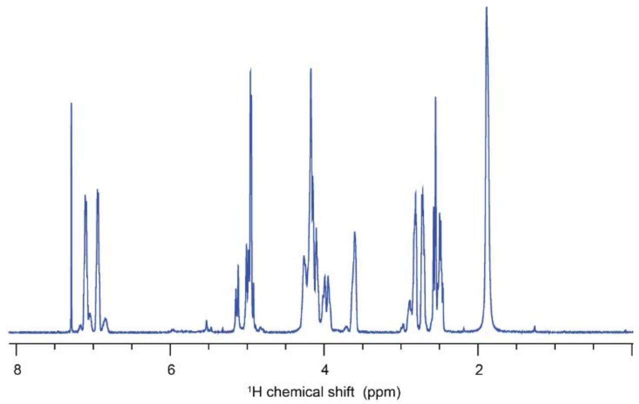

Figure S2: The ^{1}H NMR spectrum of sitagliptin collected at room temperature with $CDCl_3$ solvent.

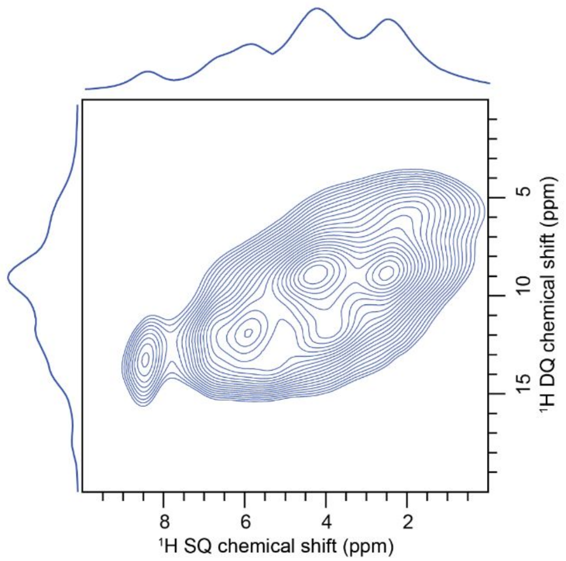

Figure S3: The ^{1}H-^{1}H BABA spectra of the sitagliptin molecule collected at spinning speed of 60 kHz.

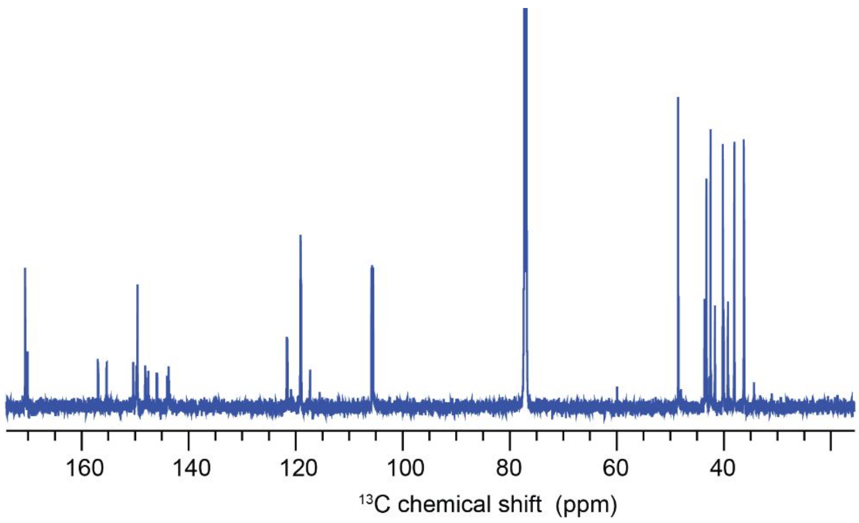

Figure S4: The ^{13}C NMR spectrum of sitagliptin collected at room temperature with $CDCl_3$ solvent.

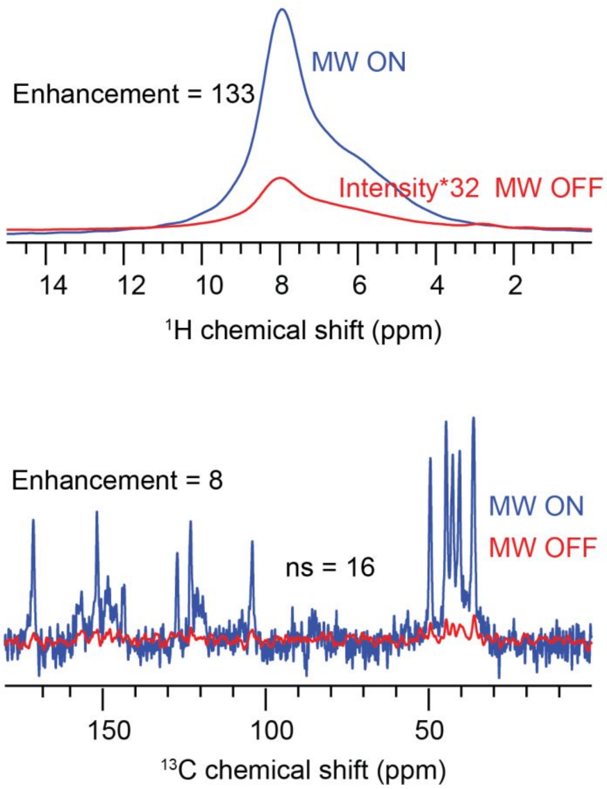

Figure S5: The DNP MW ON and OFF spectra for a) the ^{1}H and b) for the ^{13}C CP spectra collected at 10 kHz spinning speed with exogenous radical TEKPOL and LiCl salt to form the glassy matrix. An enhancement of 133 is observed for the proton whereas the enhancement of carbon is 8. The low enhancement of ^{13}C could be due to the mixture of short T_1 and spin diffusion.

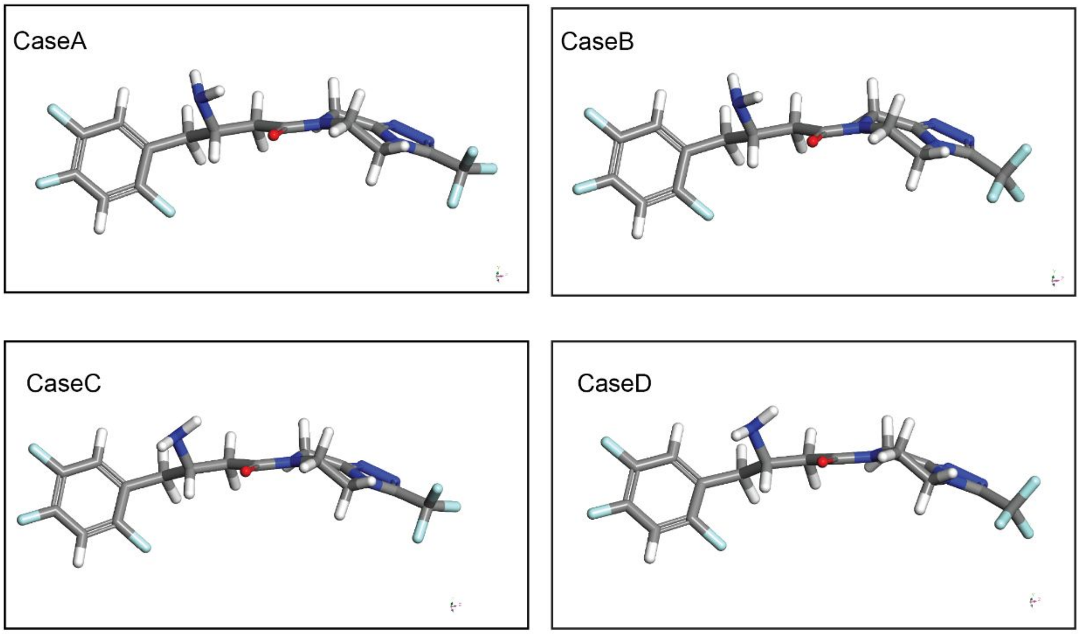

Figure S6: The structure of four different cases used in the study.

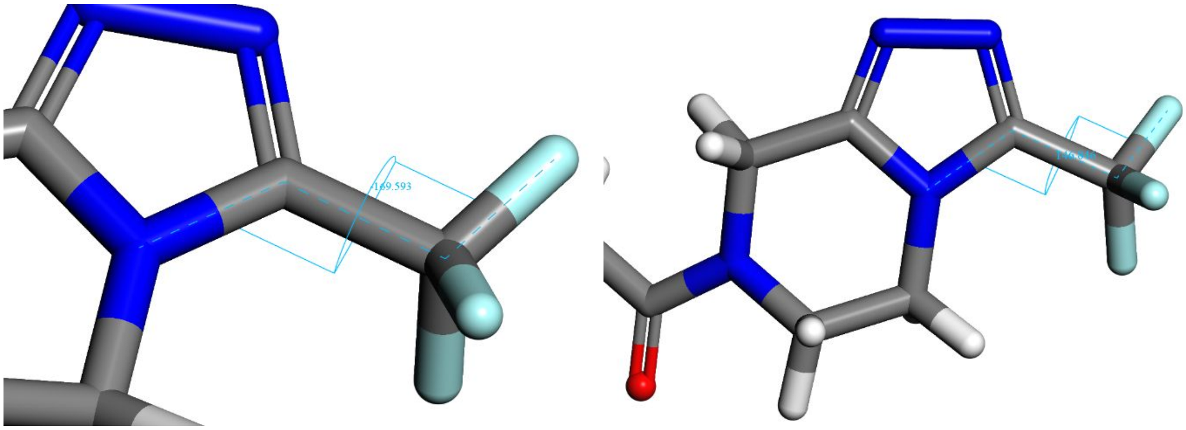

Figure S7: The orientation of CF_3 group in the case A and B.

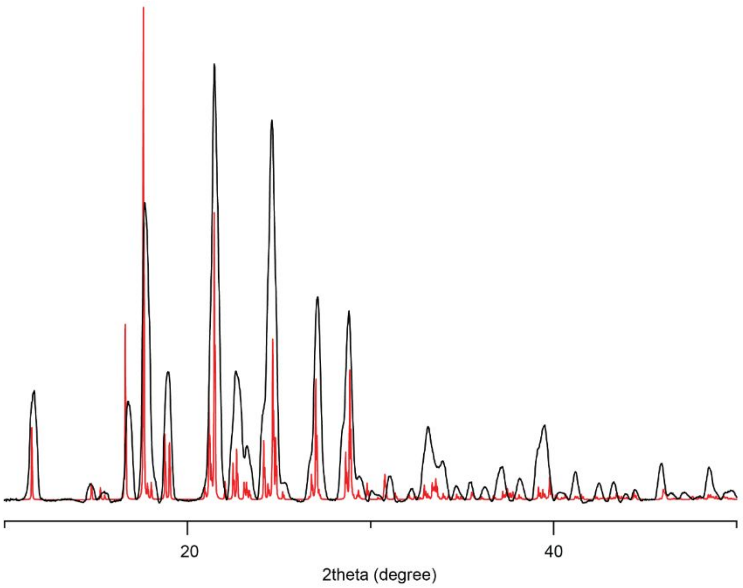

Figure S8: The comparison of the PXRD pattern from the experiment and simulation using 3D ED model.

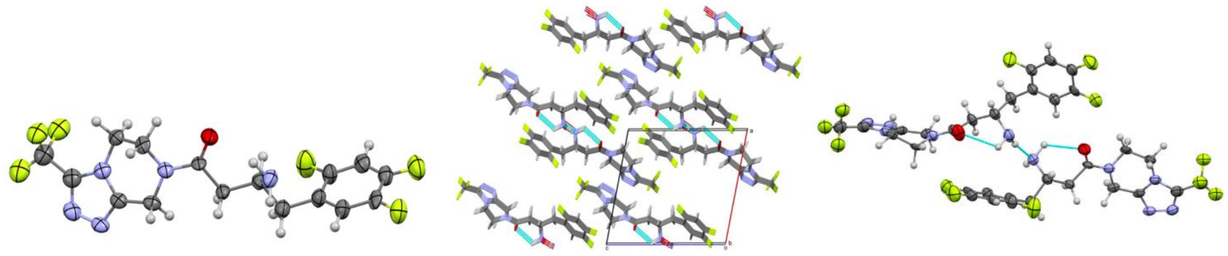

Figure S9: Structure showing the inter and intra molecular hydrogen bonding. The intramolecular hydrogen bonding occurs between the protons of NH_2 and oxygen of carbonyl group in the same molecule whereas intermolecular hydrogen bonding is between protons on the NH_2 with adjacent nitrogen. The monomer unit is oriented in such a way that the imidazole ring is perpendicular to aromatic part and it arises due to the twist arising from the aliphatic bridge.

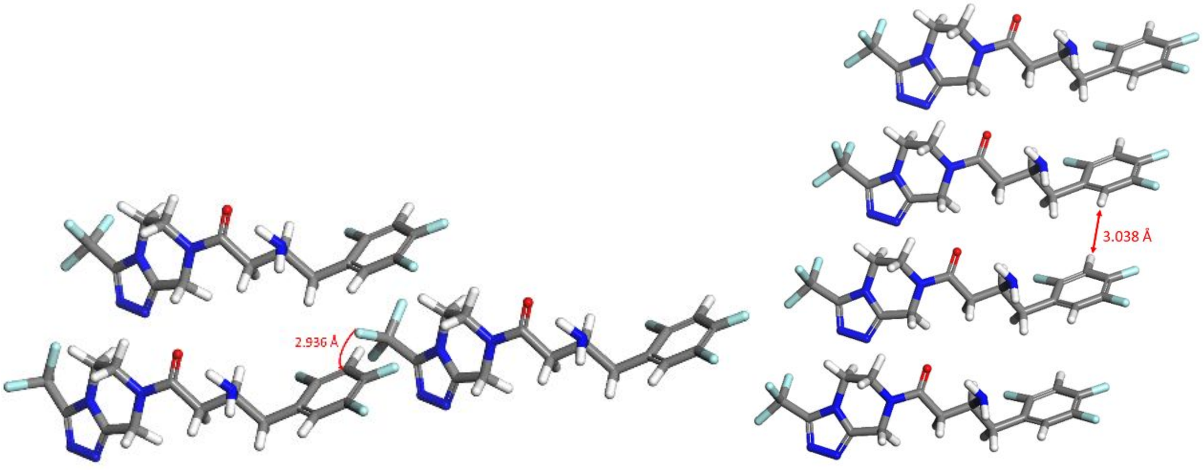

Figure S10: Schematic diagram showing the intermolecular coorelation

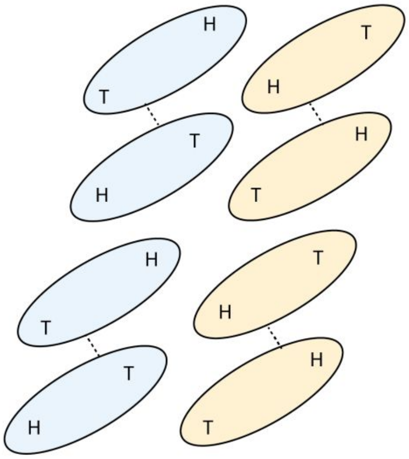

Figure S11: The schematic diagram showing the head to tail arrangement in the sitagliptin molecule.

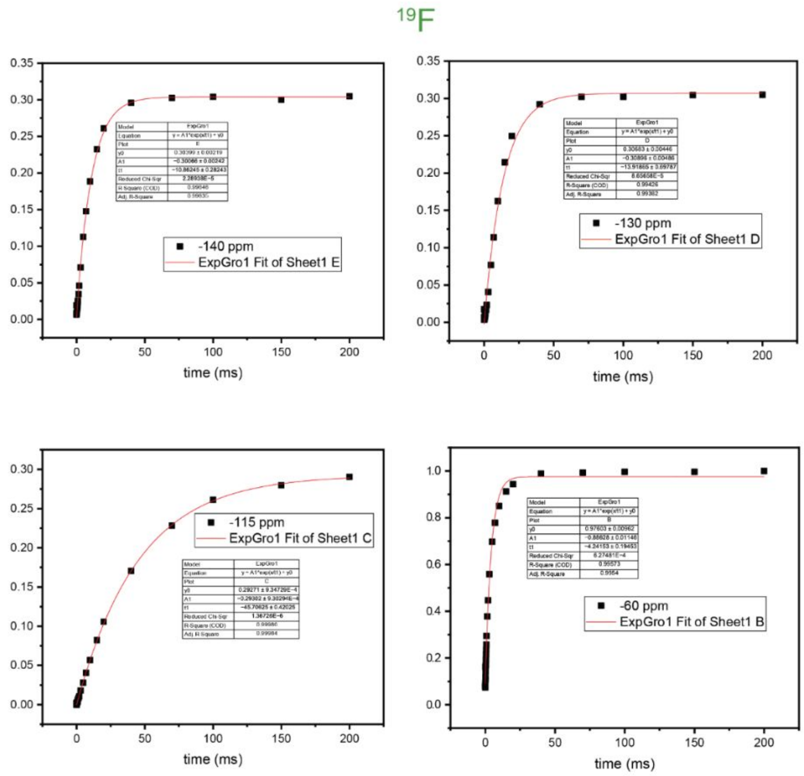

Figure S12: The T_1 relaxation curve observed for ^{19}F measured at -25 degree Celsius. The curves were fitted with monoexponential function.

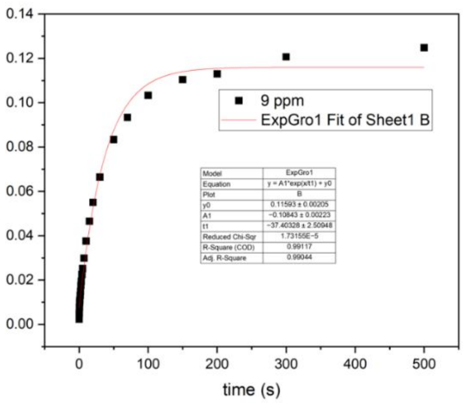

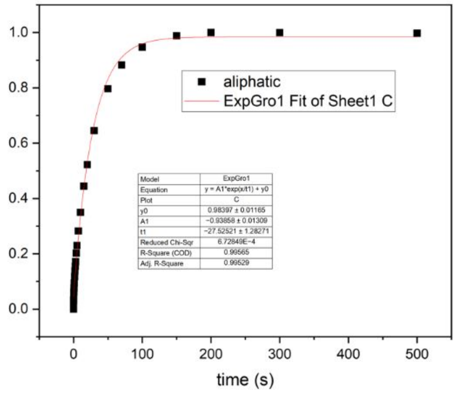

Figure S13: The T_1 build up curve observed for ^1H measured at -25 degree Celsius. The T_1 relaxation curves were fitted with mono exponential function.

Longitudinal relaxation delay of the fluorine compounds was determined by the saturation recovery pulse sequence method, using T_1/T_2 Relaxation Bruker program which fitted the data to the exponential equation:

$$I = I_0 + P\exp(-\tau/T1),$$

where I is the intensity of the compound of interest resonance at the inversion delay time τ and I_0 at the equilibrium state and P is a constant.

References

(1) Mentink-Vigier, F.; Marin-Montesinos, I.; Jagtap, A. P.; Halbritter, T.; van Tol, J.; Hediger, S.; Lee, D.; Sigurdsson, S. Th.; De Paëpe, G. Computationally Assisted Design of Polarizing Agents for Dynamic Nuclear Polarization Enhanced NMR: The AsymPol Family. *J Am Chem Soc* **2018**, *140* (35), 11013–11019. https://doi.org/10.1021/jacs.8b04911.

(2) Metz, G.; Wu, X.; Smith, S. O. Ramped-Amplitude Cross Polarization in Magic-Angle-Spinning NMR. *J Magn Reson A* **1994**, *110* (2), 219–227. https://doi.org/10.1006/jmra.1994.1208.

(3) Kolodziejski, W.; Klinowski, J. Kinetics of Cross-Polarization in Solid-State NMR: A Guide for Chemists. *Chem Rev* **2002**, *102* (3), 613–628. https://doi.org/10.1021/cr000060n.

(4) Fung, B. M.; Khitrin, A. K.; Ermolaev, K. An Improved Broadband Decoupling Sequence for Liquid Crystals and Solids. *Journal of Magnetic Resonance* **2000**, *142* (1), 97–101. https://doi.org/10.1006/jmre.1999.1896.

(5) Sakellariou, D.; Lesage, A.; Hodgkinson, P.; Emsley, L. Homonuclear Dipolar Decoupling in Solid-State NMR Using Continuous Phase Modulation. *Chem Phys Lett* **2000**, *319* (3–4), 253–260. https://doi.org/10.1016/S0009-2614(00)00127-5.

(6) Oas, T. G.; Griffin, R. G.; Levitt, M. H. Rotary Resonance Recoupling of Dipolar Interactions in Solid-state Nuclear Magnetic Resonance Spectroscopy. *J Chem Phys* **1988**, *89* (2), 692–695. https://doi.org/10.1063/1.455191.

(7) Feike, M.; Demco, D. E.; Graf, R.; Gottwald, J.; Hafner, S.; Spiess, H. W. Broadband Multiple-Quantum NMR Spectroscopy. *J Magn Reson A* **1996**, *122* (2), 214–221. https://doi.org/https://doi.org/10.1006/jmra.1996.0197.

(8) Mauri, F.; Pfrommer, B. G.; Louie, S. G. Ab Initio Theory of NMR Chemical Shifts in Solids and Liquids. *Phys Rev Lett* **1996**, *77* (26), 5300–5303. https://doi.org/10.1103/PhysRevLett.77.5300.

(9) Pickard, C. J.; Mauri, F. All-Electron Magnetic Response with Pseudopotentials: NMR Chemical Shifts. *Phys Rev B Condens Matter Mater Phys* **2001**, *63* (24), 2451011–2451013. https://doi.org/10.1103/physrevb.63.245101.

(10) Clark, S. J.; Segall, M. D.; Pickard, C. J.; Hasnip, P. J.; Probert, M. I. J.; Refson, K.; Payne, M. C. First Principles Methods Using CASTEP. *Zeitschrift fur Kristallographie* **2005**, *220* (5–6), 567–570. https://doi.org/10.1524/zkri.220.5.567.65075.

(11) Perdew, J. P.; Ruzsinszky, A.; Csonka, G. I.; Vydrov, O. A.; Scuseria, G. E.; Constantin, L. A.; Zhou, X.; Burke, K. Restoring the Density-Gradient Expansion for Exchange in Solids and Surfaces. *Phys Rev Lett* **2008**, *100* (13), 136406. https://doi.org/10.1103/PhysRevLett.100.136406.

(12) Yates, J. R.; Pickard, C. J.; Mauri, F. Calculation of NMR Chemical Shifts for Extended Systems Using Ultrasoft Pseudopotentials. *Phys Rev B Condens Matter Mater Phys* **2007**, *76* (2). https://doi.org/10.1103/PhysRevB.76.024401.

(13) Tkatchenko, A.; Scheffler, M. Accurate Molecular van Der Waals Interactions from Ground-State Electron Density and Free-Atom Reference Data. *Phys Rev Lett* **2009**, *102* (7). https://doi.org/10.1103/PhysRevLett.102.073005.

(14) Monkhorst, H. J.; Pack, J. D. Special Points for Brillouin-Zone Integrations. *Phys Rev B* **1976**, *13* (12), 5188–5192. https://doi.org/10.1103/PhysRevB.13.5188.

(15) ELDIX Software Suite Version 0.14.0; ELDICO Scientific AG. ELDIX Software Suite, Version 0.14.0; ELDICO Scientific AG: Villigen, Switzerland, 2022. Villigen 2022.

(16) Apex Suite of Crystallographic Software APEX4 Version 2022; Bruker AXS Inc. Apex Suite of Crystallographic Software, APEX4, Version 2022.1-1; Bruker AXS Inc.: Madison, WI, USA, 2022. Madison 2022.

(17) SADABS; Version 2016/2 Bruker AXS Inc. SADABS, Version 2016/2; Bruker AXS Inc.: Madison, WI, USA, 2016. Madison 2016.

(18) SAINT Version 8.40 B Bruker AXS Inc. SAINT, Version 8.40B; Bruker AXS Inc.: Madison, WI, USA, 2019. Bruker AXS Inc: Madison 2019.

(19) Sheldrick, G. M. Crystal Structure Refinement with SHELXL. *Acta Crystallographica Section C* **2015**, *71* (1), 3–8. https://doi.org/10.1107/S2053229614024218.

(20) Hübschle, C. B.; Sheldrick, G. M.; Dittrich, B. ShelXle: A Qt Graphical User Interface for SHELXL. *J Appl Crystallogr* **2011**, *44* (6), 1281–1284. https://doi.org/10.1107/S0021889811043202.
(21) Mathew, R.; Mazumder, A.; Kumar, P.; Matula, J.; Mohamed, S.; Brazda, P.; Hariharan, M.; Thomas, B. Unveiling the Topology of Partially Disordered Micro-Crystalline Nitro-Perylenediimide with X-Aggregate Stacking: An Integrated Approach. *Chem. Sci.* **2024**, *15* (2), 490–499. https://doi.org/10.1039/D3SC05514K.
(22) Sasikumar, D.; Vinod, K.; Sunny, J.; Hariharan, M. Exciton Interactions in Helical Crystals of a Hydrogen-Bonded Eumelanin Monomer. *Chem Sci* **2022**, *13* (8), 2331–2338. https://doi.org/10.1039/D1SC06755A.
(23) Guzmán-Afonso, C.; Hong, Y.; Colaux, H.; Iijima, H.; Saitow, A.; Fukumura, T.; Aoyama, Y.; Motoki, S.; Oikawa, T.; Yamazaki, T.; Yonekura, K.; Nishiyama, Y. Understanding Hydrogen-Bonding Structures of Molecular Crystals via Electron and NMR Nanocrystallography. *Nat Commun* **2019**, *10* (1), 3537. https://doi.org/10.1038/s41467-019-11469-2.
(24) Smalley, C. J. H.; Hoskyns, H. E.; Hughes, C. E.; Johnstone, D. N.; Willhammar, T.; Young, M. T.; Pickard, C. J.; Logsdail, A. J.; Midgley, P. A.; Harris, K. D. M. A Structure Determination Protocol Based on Combined Analysis of 3D-ED Data, Powder XRD Data, Solid-State NMR Data and DFT-D Calculations Reveals the Structure of a New Polymorph of l-Tyrosine. *Chem Sci* **2022**, *13* (18), 5277–5288. https://doi.org/10.1039/D1SC06467C.
(25) Thomas, B.; Rombouts, J.; Oostergetel, G. T.; Gupta, K. B. S. S.; Buda, F.; Lammertsma, K.; Orru, R.; de Groot, H. J. M. A Hybrid Solid-State NMR and Electron Microscopy Structure-Determination Protocol for Engineering Advanced Para-Crystalline Optical Materials. *Chemistry – A European Journal* **2017**, *23* (14), 3280–3284. https://doi.org/https://doi.org/10.1002/chem.201700324.
